# Seasonal Changes in Plankton Food Web Structure and Carbon Dioxide Flux from Southern California Reservoirs

**DOI:** 10.1371/journal.pone.0140464

**Published:** 2015-10-16

**Authors:** Emily M. Adamczyk, Jonathan B. Shurin

**Affiliations:** Division of Biological Sciences, Section of Ecology, Behavior and Evolution, University of California San Diego, La Jolla, California, United States of America; University of Shiga Prefecture, JAPAN

## Abstract

Reservoirs around the world contribute to cycling of carbon dioxide (CO_2_) with the atmosphere, but there is little information on how ecosystem processes determine the absorption or emission of CO_2_. Reservoirs are the most prevalent freshwater systems in the arid southwest of North America, yet it is unclear whether they sequester or release CO_2_ and therefore how water impoundment impacts global carbon cycling. We sampled three reservoirs in San Diego, California, weekly for one year. We measured seasonal variation in the abundances of bacteria, phytoplankton, and zooplankton, as well as water chemistry (pH, nutrients, ions, dissolved organic carbon [DOC]), which were used to estimate partial pressure of CO_2_ (pCO_2_), and CO_2_ flux. We found that San Diego reservoirs are most often undersaturated with CO_2_ with respect to the atmosphere and are estimated to absorb on average 3.22 mmol C m^-2^ day^-1^. pCO_2_ was highest in the winter and lower in the summer, indicating seasonal shifts in the magnitudes of photosynthesis and respiration associated with day length, temperature and water inputs. Abundances of microbes (bacteria) peaked in the winter along with pCO_2_, while phytoplankton, nutrients, zooplankton and DOC were all unrelated to pCO_2_. Our data indicate that reservoirs of semi-arid environments may primarily function as carbon sinks, and that carbon flux varies seasonally but is unrelated to nutrient or DOC availability, or the abundances of phytoplankton or zooplankton.

## Introduction

Feedbacks between ecosystems and the atmosphere determine the fate of anthropogenic CO_2_ and the potential for biological sequestration. The physical and biological processes that occur within lakes determine the metabolic balance between respiration and photosynthesis, and therefore whether they sequester or emit CO_2_ [1]. Since lakes are generally at low positions on the landscape, they often receive large quantities of terrestrial detritus which is either respired, generating CO_2_ that escapes to the atmosphere, or stored in the sediments. Lakes with high concentrations of allochthonous dissolved organic carbon (DOC) are therefore often net sources of CO_2_ [2–4]. However, processes that affect in situ primary productivity may affect the rate of biological CO_2_ uptake by autotrophs. The supply of inorganic nutrients and rate of grazing by heterotrophic consumers can play a large role in carbon exchange. Eutrophic lakes with very high productivity are often CO_2_ depleted [5,6]. In addition, predators can indirectly control primary production through trophic cascades, resulting in greater CO_2_ uptake when herbivores are under strong top-down control [1,7]. The relative roles of local ecological interactions vs. processes in the watershed or the atmosphere that determine the supply of allochthonous organic material are poorly understood.

Manmade reservoirs contribute about 37% of the global efflux of CO_2_ to the atmosphere from all freshwater ecosystems, including lakes, rivers, streams, groundwater, and brackish ecosystems such as wetlands and estuaries [8]. When a landscape is flooded to create a reservoir, terrestrial plant communities are inundated and organic material may either accumulate in sediments or decompose, releasing CO_2_ [9]. The decrease in terrestrial photosynthesis and assimilation of CO_2_ may result in the loss of an atmospheric CO_2_ sink [10]. The sequestration of organic carbon in inland water sediments is estimated to exceed the global burial of organic carbon on the ocean floor three-fold [11,12]. It is also estimated that artificial reservoirs emit twice the quantity of CO_2_ compared to natural lakes [8,13]. Studies have shown that in a newly created reservoir, greenhouse gas (GHG) emissions, including CO_2_ and CH_4_, generally increase rapidly within a period of 10 years, after which emission rates decrease and become equivalent to those found in natural lakes [14]. Metabolism of organic carbon compounds by aquatic bacteria in reservoirs can exceed autotroph photosynthesis, resulting in a net source of CO_2_ to the atmosphere [15]. The effect of impoundment on GHG fluxes is the difference between the fluxes of those emissions before and after flooding [13]. The role of reservoirs in the global carbon cycle is therefore uncertain but potentially significant.

**Fig 1:**
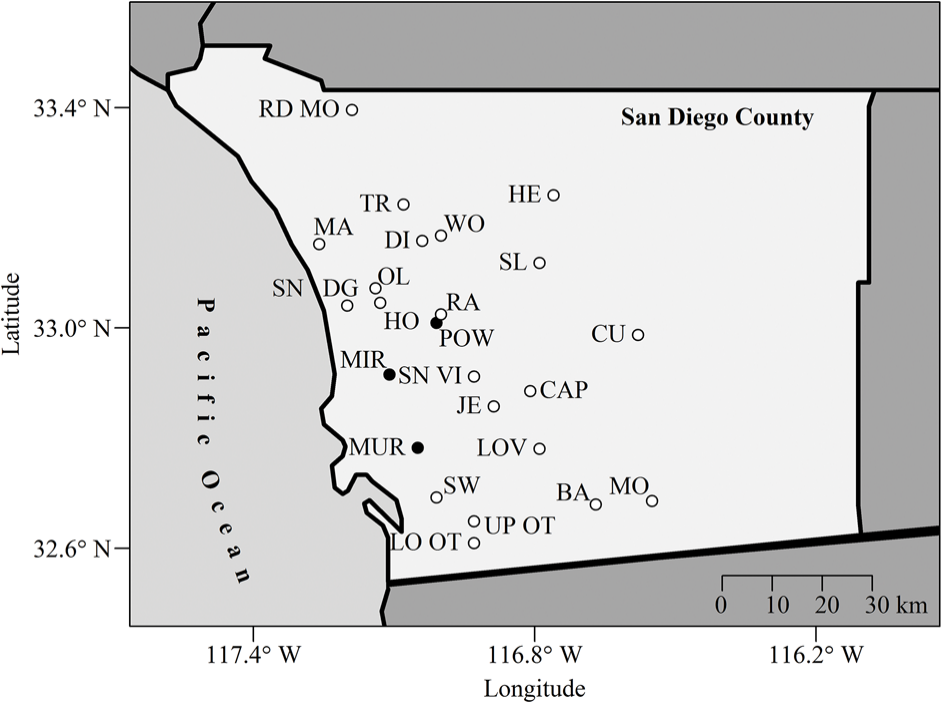
Twenty-four major reservoirs in San Diego County, California, USA http://www.sdcwa.org/san-diego-county-water-sources (http://www.sdcwa.org/reservoirs). Barret (BA); Cuyamaca (CU); Dixon (DI); El Capitan (CAP); Henshaw (HE); Hodges (HO); Jennings (JE); Loveland (LOV); Lower Otay (LO); Maerkle (MA); **Miramar (MIR)**; Morena (MO); **Murray (MUR)**; Olivenhain (OL); **Poway (POW)**; Ramona (RA); Red Mountain (RD MO); San Dieguito (SN DG); San Vicente (SN VI); Sutherland (SL); Sweetwater (SW); Turner (TR); Upper Otay (UP OT); Wohlford (WF). **The black points indicate the three reservoirs we sampled.**

Reservoirs for water storage have been extensively constructed throughout the arid southwest of North America, and represent the largest area of freshwater ecosystems. In San Diego County, most of freshwater for human use is imported. From 2009 to 2013, the five-year average imported water supplies for San Diego County included 63% from the Colorado River, 20% from the Sacramento and San Joaquin rivers, and the last 17% from local supplies including runoff from seven watersheds, groundwater, and recycled waste water (http://www.sdcwa.org/imported-supplies). Twenty-four major reservoirs in the county (Fig 1) store water for human consumption and are used for recreational activities such as boating and fishing. There are no data on the CO_2_ budgets of these reservoirs. Additionally, no studies have investigated how the transport of water over great distances and its storage affects the carbon cycle in Southern California. With the enactment of California’s Assembly Bill 32 in 2006, many counties have been taking steps to reduce GHG emissions, including investigating the impacts of reservoirs on CO_2_ and CH_4_ [16]. As water demand increases with continued human population growth, resulting in more construction of dams, it is important to understand the impact of creating and maintaining reservoirs on the global carbon cycle.

Although tropical and temperate reservoirs function mainly as net sources of CO_2_ to the atmosphere [17,18], the few studies of the carbon balance of western United States (U.S.) reservoirs have reached contradictory conclusions. Therrien et al. [19] found that 46 reservoirs in semi-arid ecosystems throughout the southwestern U.S. (Arizona, New Mexico, and Utah) were super-saturated with CO_2_. In contrast, a study on GHG emissions from six reservoirs throughout Northern California, Oregon, Washington, and Idaho showed that four were sinks for atmospheric CO_2_ [20]. Neither of these studies examined temporal variation in net CO_2_ flux in reservoirs or its relationship to biological or physical processes. Morales-Pineda et al. [21] found that two stratified reservoirs in a semi-arid region of Spain were super-saturated with CO_2,_ and that pCO_2_ showed considerable temporal variability. Thus, temporal variation in the carbon balance of reservoirs, and their relationship to ecosystem structure, remain in question.

Net CO_2_ flux between aquatic ecosystems and the atmosphere is determined primarily by inputs of organic matter from the watershed, the physical processes that regulate gas exchange and biological processes that determine in situ rates of photosynthesis and respiration. Wind, water temperature, vertical stratification and concentrations of solutes all affect gas exchange. Dissolved organic carbon (DOC) can also drive high rates of respiration by providing substrate for the growth of aquatic heterotrophic bacteria [11,22,23]. CO_2_ supersaturation in lakes and its emission to the atmosphere has been shown to be a result of allochthonous DOC which drives net heterotrophy and bacteria metabolism [22,24,25]. Finally, conditions that favor increased autotrophic production, including high nutrient supply or reduced top-down control by grazers, can increase CO_2_ uptake and sequestration [1,6,26]. The balance of these processes in reservoirs of the semi-arid south west of North America, and their seasonal variability and relationship to food web structure, are unknown.

The goal of this study was to elucidate how seasonal changes in abiotic conditions, food web structure, and the supply of organic and inorganic resources determine the net balance of CO_2_ flux between reservoirs and the atmosphere. We addressed two questions: (1) Do reservoirs in semi-arid regions such as San Diego County sequester or emit atmospheric CO_2_? (2) How do ecosystem processes regulate variation in CO_2_ flux/pCO_2_ in these reservoirs in space and time? To address these questions, we sought to identify the key biotic and abiotic drivers for variation among reservoirs and over time in pCO_2_. We sampled three reservoirs in San Diego (Murray, Miramar, and Poway) weekly for the partial pressure of CO_2_, water chemistry, vertical stratification, wind speed, and the abundances of phytoplankton, bacteria, and zooplankton in the water column. Our data allowed us to determine how seasonal variation in ecological processes and species interactions influence absorption of atmospheric carbon.

## Materials and Methods

We sampled three reservoirs in San Diego County: Lake Miramar, Lake Murray, and Lake Poway on a weekly basis for a total of 50 times between 26 June 2013 and 26 June 2014 (Table 1). All three reservoirs contained both piscivorous and zooplanktivorous fish, primarily bass, trout, and catfish that are stocked annually for sport fisheries. We sampled zooplankton, phytoplankton, bacteria, and measured total alkalinity, concentrations of total phosphorous and nitrogen, water and air temperature, pH, partial pressure of CO_2_, dissolved oxygen, dissolved organic carbon, particulate organic carbon and nitrogen, and wind speed. Reservoirs were sampled at the deepest point from a small boat. Permission was not required on public waterways (Lakes Miramar, Murray, and Poway) used for recreation and fishing. These studies did not involve endangered or protected species in any way.

**Table 1:** Characteristics of three reservoirs sampled weekly from 26 June 2013–26 June 2014.

Reservoir	Lake Miramar	Lake Murray	Lake Poway
Location	32°92’N; 117°10’W	32°78’N; 117°04’W	33°01’N; 117°01’W
Elevation, (m)	215	162	285
Year Built	1960	1918	1971
Surface Area, (km^2^)	0.660	0.809	0.263
Maximum Volume, (m^3^)	8.24 x 10^6^	5.78 x 10^6^	4.11 x 10^6^
Maximum Depth, (m)	35	29	36
Weather Station Location	32°86’N; 117°14’W	32°83’N; 116°97’W	33°00’N; 117°04’W

### Water Chemistry and Organism Collection

A YSI® multiparameter sonde (Xylem Inc.: Professional Plus) was used to measure vertical profiles of water and air temperature (°C), dissolved oxygen (mg L^-1^), and pH. The data were recorded throughout the water column every 4 m up to a maximum depth of 16–17 m in Lake Murray and 20 m in Lakes Miramar and Poway. We did not sample Lake Murray at 20 m depth because the deepest part of the reservoir was inaccessible by our boat. The pH probe was calibrated with standard solutions before sampling. When the field sonde was not available for use (17 sampling dates), a HOBO Pendant® Temperature/Light Data Logger (Onset®: 8K –UA-002-08) was used to measure water and air temperature (°C) and water was collected from a depth of 1 m to analyze pH in the lab. On these occasions, pH was measured using a VWR® SympHony™ SB80PC Benchtop Meter. The pH probe was pre-calibrated with pH standards of 4, 7, and 10 before analyzing samples.

Surface water samples were collected for measuring phytoplankton chlorophyll-*a* concentration, total nitrogen and phosphorus concentrations, abundances of bacteria, alkalinity, and particulate organic carbon (POC) and nitrogen (PON) with a 1 m integrated tube sampler. Water samples were filtered through a 63 μm sieve to remove zooplankton, and sample bottles were pre-rinsed with lake water. pCO_2_ was determined based on measured values of water temperature, total alkalinity, pH, and total phosphorus using the model CO2calc 1.2.0 [27]. Parameter values for CO2calc were assigned as follows: salinity was 0 ppt for freshwater; the constants K1 and K2 were from Millero [28], the acid dissociation constant of bisulfate ion was from Dickson [29], and pH was used on a NBS scale (mol kg^-1^ water). The details of the model for calculation of pCO_2_ are described in [27].

### Wind Speed

On each sampling day, average wind speed from weather stations (http://www.wunderground.com/) near each reservoir was recorded and used in calculating CO_2_ flux (Table 1).

### CO_2_ Flux

CO_2_ flux was calculated using water temperature, total phosphorus, total alkalinity, pH, atmospheric partial pressure of carbon dioxide from Mauna Loa Observatory in Hawaii, USA (https://twitter.com/Keeling_curve), and wind speed. For weeks where there were no atmospheric pCO_2_ data, concentrations of CO_2_ were averaged from the previous and following weeks. CO2calc 1.2.0 was used to obtain CO_2_ flux values [27].

### Phytoplankton (chlorophyll-*a*) Biomass

To measure in vivo chlorophyll-*a* (chl-*a*) concentrations, we collected surface water from 1 m depth at each reservoir using an integrated tube sampler. The samples were filtered through a 63 μm sieve to remove any zooplankton and were stored in plastic bottles that were pre-rinsed with lake water. To standardize our measurements of chl-*a*, we kept the sample bottles in a dark cooler for no more than 2–4 hours to before analysis [30,31]. In the laboratory, we mixed our samples by inverting the plastic bottles to prevent settlement of phytoplankton cells. We quickly pipetted 1600 μL of the surface water into glass vials and ran the samples through a pre-calibrated Turner Trilogy® Laboratory Fluorometer. We used the chlorophyll-*a* fluorescence in vivo module (P/N 7200–043), which had an emission wavelength of 660–710 nm and an excitation wavelength of 485 nm, which were appropriate for measuring in vivo chl-*a* concentrations [31,32].

### Total Nitrogen and Phosphorous

Water collected for total nitrogen and phosphorus analysis was preserved in 20 mL scintillation vials which were rinsed three times with reservoir water before being filled. For the first 25 sampling dates, nutrient samples were preserved with 400 μL H_2_SO_4_, but after the 25^th^ date, the acidity was reduced to 40 μL H_2_SO_4_ since the lowered acidity was found to provide more accurate nutrient analysis results. The samples with a lowered acidity had mean TN and TP concentrations that were respectively 0.02 and 0.03 mg L^-1^ greater than the more acidic samples. These differences are small relative to the mean and range of values across our data set, therefore the change in methodology likely had minimal impact on our results. The samples were frozen at -26.5°C until analysis. To measure total nitrogen and phosphorous concentrations, the QuikChem® Method 10-107-04-4-C and QuikChem® Method 10-115-01-4-C were used respectively on a QuikChem® 8500 Series 2 Flow Injection Analysis System (Lachat Instruments). The data were analyzed using Omnion 3.0 software (Lachat: Hach 2007).

### Bacteria Abundance

To measure abundances of pelagic bacteria, 10 mL of surface water from each reservoir was collected in pre-rinsed 20 mL scintillation vials. The samples were preserved with 1 mL 20% phosphate buffered saline (PBS) buffered formaldehyde fixing agent. PBS solution was made by dissolving 0.69g NaH_2_PO_4_, 1.34g Na_2_HPO_4_ and 7.6g NaCl in 1L deionized (DI) water. For making 1 L 20% phosphate buffered saline (PBS) buffered formaldehyde fixing agent, 540.5 mL of 37% formaldehyde was added to 459.5 mL PBS buffer solution and was stored in a sterile container. Each sample was mixed thoroughly and stored at room temperature for no longer than 2 weeks before counting cells by epifluorescence microscopy. Bacteria were stained with DAPI (4',6-diamidino-2-phenylindole) fluorescent stain [33]. Brown track-etched poly carbonate membrane filters (Millipore® GTBP02500 Isopore™; 0.22 μm porosity, 25 mm diameter) for bacteria were rinsed with sterile DI water prior to use. Filters were placed on a vacuum filtration manifold (Millipore® 1225) and a maximum of 2 mL of lake water was added. If the concentration of the sample was too dense, 0.5–1.5 mL of sample was added and the rest was filled with sterile deionized (DI) water that had been autoclaved at 121°C and 17 psi for 15 minutes (Tuttnauer®). The manifold well was shaken to disperse the sample evenly and 25 μL DAPI stain (1.25% of sample) was added for staining bacteria. The filters were covered to block any light and samples were stained for 20 minutes.

After 20 minutes, solution on top of the filters were removed and any unincorporated stain was rinsed off with 5 mL of sterile DI water. The filters were placed on top of one drop of non-fluorescent immersion oil on a clean microscope slide. One more drop of non-fluorescent immersion oil was placed on top of the filters and they were mounted with a coverslip. The slides were kept in the dark until they were counted in a dark room on an OMAX® EPI-fluorescence microscope (40X-1000X Top Quality Professional Infinite) within 1 hour. A 100X objective lens (0.22 mm in diameter) was used to count bacteria and at least 10 different fields of view of the filter sample were counted. The average number of bacteria per counting field were calculated and then multiplied by the ratio of the area of filter to area visible under magnification to obtain the total number of bacteria per liter.

### Total Alkalinity

Alkalinity was measured by adding 2 mL of Orion™ total alkalinity solution to 20 mL of water sample. The pH of the sample was measured using a VWR® SympHony™ SB80PC Benchtop Meter pH probe, which was pre-calibrated with pH standards of 4, 7, and 10, and the resulting value was converted to alkalinity using an Orion™ Total Alkalinity Test Kit converter wheel.

### Particulate Organic Carbon (POC) and Nitrogen (PON)

Water for POC and PON was filtered through either a 47 mm or 25 mm Whatman® glass microfiber filter (GF/F: 0.7 μm porosity), which had been pre-combusted in a drying oven at 500°C for at least four hours. The filter was placed on a Büchner funnel on top of a flask and between 200 mL–1 L of water sample was filtered depending on the productivity of the reservoirs. The filters were individually wrapped in aluminum foil and frozen at -20.5°C until analysis. Samples were freeze-dried at -70°C for 24 hours. Before analysis, filters were cut into quarters in a sterile environment, folded up in 9x10 mm aluminum tins (Costech Analytical Technologies Inc.) and desiccated until analysis. The samples were analyzed for carbon/nitrogen content using a ECS 4010 CHNSO Analyzer (Costech Analytical Technologies, Inc.) at Scripps Institution of Oceanography. The elemental analysis ran under Costech software: semimicro amount of oxygen was used during a 5 minute total run time and water was removed. Five standards and a tin blank were run, spaced evenly throughout the samples. The standards were plotted versus weight and a linear fit was determined for calibration. Particulate organic carbon and nitrogen concentrations were determined.

### Dissolved Organic Carbon

Water for dissolved organic carbon (DOC) analysis was collected at 1 m depth, filtered through a Whatman® 25 mm GF/F (0.7 μm porosity) which was pre-combusted in a drying oven at 500°C for at least four hours, and stored in pre-rinsed 40 mL vials (VWR®TraceClean®). The samples were preserved with 250 μL trace metal grade hydrochloric acid and analyzed with a Shimadzu® TOC-V CSN at Scripps Institution of Oceanography.

### Zooplankton

Zooplankton were collected throughout the water column using a 63 μm mesh net (30 cm in diameter) lowered to 2 m above the bottom. One tow was sampled per lake per week resulting in 146 total samples. Zooplankton were preserved with 70% ethyl alcohol: zooplankton were counted by splitting samples using a Wildco® Folsom Plankton Splitter (0.5 L) and at least 179 animals total ($\bar{X}$ = 331; *range* = 179–969) were counted for each aliquot using a Leica M125 stereo microscope. Copepods were counted by order and developmental stages while cladocerans were identified to species. To measure body size of zooplankton in order to estimate population weight, at least 25 animals per sample of each species were photographed using a Leica DFC295 camera and Leica V3.5.0 software. When fewer than 25 animals of a species were present in a sample, all individuals were measured. Zooplankton lengths were measured using ImageJ [34] from digitized analysis of calibrated images, and length-weight regression equations from [35–40] were used to calculate mean individual mass and population biomass.

### Statistical Analyses

For each variable, a two-way analysis of variance (ANOVA) was run with lakes and dates as predictors to determine which variables varied in space or time. Multiple regression models including all candidate predictor variables sampled were run to determine main drivers of pCO_2_ and pH across all reservoirs. Log transformations were applied to independent variables to normalize variance as necessary, and second order terms were included to test for non-linear relationships. The variables included in the base model to predict pCO_2_ were: log(POC), log(PON), log(TN), log(TP), log(chl-*a*), log(zooplankton community biomass), log(bacteria), and log(DOC). These variables were used to identify the primary drivers of pCO_2_. “Lake” was included as a random variable in the model, and a first order autoregressive term was included. A model averaging procedure based on all possible submodels including the independent variables (the function “dredge” in the package MuMIn in R [41] was used to identify the variables included in the most likely model. Since pCO_2_ was calculated based on water temperature, pH, total phosphorus, and total alkalinity, we excluded these variables from the independent variables in the candidate models to predict pCO_2_. All statistical analyses were performed in R v. 0.99.691 [42].

## Results

### Seasonal Patterns in pCO_2_ and pH

**Fig 2:**
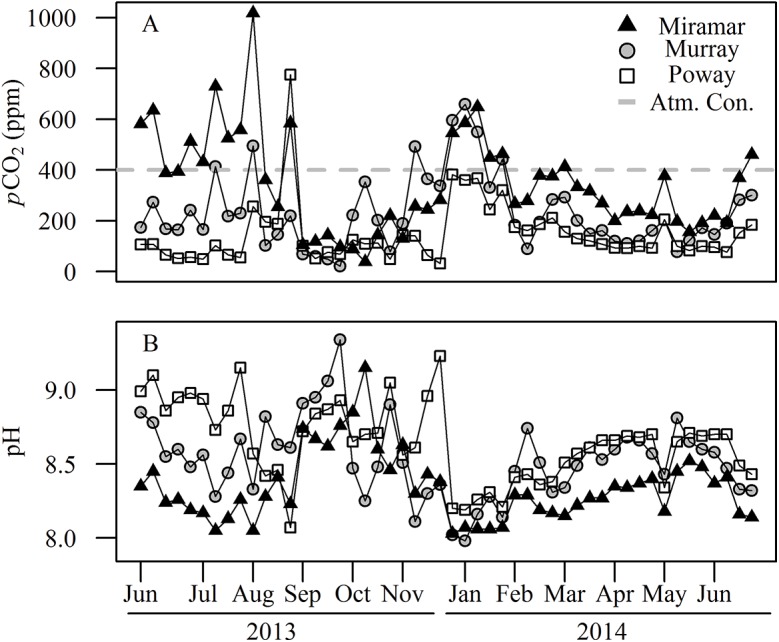
pCO2 and pH time series. (A) Time series of partial pressure of carbon dioxide (pCO_2_) in ppm for each of the three reservoirs. Current atmospheric concentrations of pCO_2_ are ~400ppm. (B) pH for each reservoir for 50 sampling weeks. Symbols indicate the reservoir as shown in the legend.

We found that pCO_2_ in all reservoirs was primarily undersaturated relative to atmospheric pCO_2_ and was significantly different between reservoirs (*F*_2,141_; *p* < 0.001). pCO_2_ concentrations increased during the winter and decreased during summer months in the three reservoirs (Fig 2A), with the exception that Lake Miramar was supersaturated with CO_2_ during much of June and July of 2013. pCO_2_ was often below 100 ppm in the summer, and was typically higher in Lake Murray ($\bar{X}$ = 232 ppm) and Miramar ($\bar{X}$ = 345 ppm) than in Lake Poway ($\bar{X}$ = 149 ppm). Lake Miramar showed high pCO_2_ during the summer of 2013 before declining around August, and then rising again in the winter months and declining in the following summer. The other two reservoirs showed more consistent patterns of low pCO_2_ during the two summers and higher in the winter. The mean pCO_2_ for all reservoirs was 241.95 ppm.

pH exhibited a seasonal trend of declining values during the winter months and increased during the summer, consistent with increased net ecosystem production in the warm seasons and greater respiration in the winter (Fig 2B). Additionally, pH was inversely correlated with pCO_2_ (*r* = -0.810). pH was generally highest in Lake Poway ($\bar{X}$ = 8.649), lowest in Lake Miramar ($\bar{X}$ = 8.339), and intermediate in Lake Murray ($\bar{X}$ = 8.530). The average pH across all reservoirs was 8.51.

### pCO_2_ and pH Models

**Fig 3:**
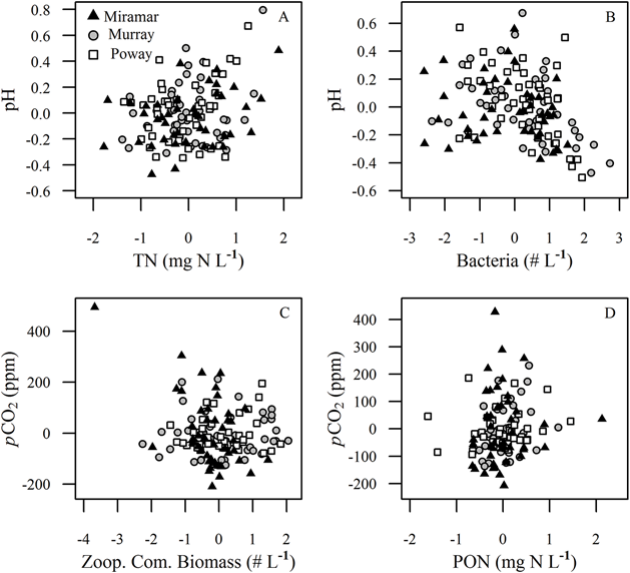
Partial regression plots of the significant drivers of pH and pCO_2_ across all reservoirs. Drivers were identified by the model averaging procedure pH versus (A) total nitrogen (mg N L^-1^) and (B) bacteria abundance (# L^-1^), and pCO_2_ versus (C) zooplankton community biomass (mg L^-1^) and (D) particulate organic nitrogen (mg N L^-1^). Symbols indicate the reservoir as shown in the legend.

The model to predict pCO_2_ based on the variables used to calculate it (water temperature, total alkalinity, pH, and total phosphorus) identified pH as the dominant factor determining pCO_2_. pH explained 64% of the variation in pCO_2,_ while the other variables explained only between <1% (total phosphorus) and 17% (total alkalinity) of the variation. We therefore constructed a model to predict pH based on the other bio-physical variables that were not included in the calculation of pCO_2_. The variables included in the top six models to predict pH were TN and bacteria (Fig 3A and 3B). Chlorophyll-*a*, POC, PON, and DOC were also included in the best six models to predict pH, but none contributed significantly to the fit of the data (Table 2A). The most important variables to predict pCO_2_ identified by the model averaging were zooplankton biomass and PON (Fig 3C and 3D). Other variables included in the top six best fit models to predict pCO_2_ included TN, bacterial abundance, and chlorophyll-*a*; however, none of these contributed significantly to the fit of the model (Table 2B). Zooplankton biomass and PON were not correlated in our data set (Pearson correlation = 0.024, *p* = 0.779).

**Table 2:** Variables included in the six top models to predict pH and pCO_2_.

**A**					
**Coefficient**	**Estimate**	**Std. Error**	***z*-value**	***p*-value**	**Importance**
log(bacteria)	-0.05689	0.02487	2.226	0.02346 *	1.00
log(TN)	0.08149	0.02539	3.179	0.00148 **	1.00
log(POC)	-0.04611	0.04532	1.014	0.31066	0.63
log(chl-*a*)	-0.02732	0.04409	0.617	0.53744	0.43
log(PON)	-0.02525	0.03885	0.648	0.51681	0.37
log(DOC)	0.05076	0.13892	0.363	0.71628	0.23
**B**					
**Coefficient**	**Estimate**	**Std. Error**	***z*-value**	***p*-value**	**Importance**
log(PON)	35.302	15.284	2.288	0.0221 *	1.00
log(zoop.)	-17.639	9.689	1.804	0.0713 .	1.00
log(TN)	-8.048	11.551	0.694	0.4879	0.48
log(bacteria)	5.245	9.881	0.529	0.5971	0.33
log(chl-*a*)	6.843	15.858	0.430	0.6675	0.26

Models to predict (A) pH and (B) pCO_2_ were based on the “dredge” procedure in the R package MuMIn [41]. The Importance value is the sum of Akaike weights over all models containing the explanatory variable.

. *p* > 0.05

* *p* < 0.05

** *p* < 0.01.

### CO_2_ Flux

**Fig 4:**
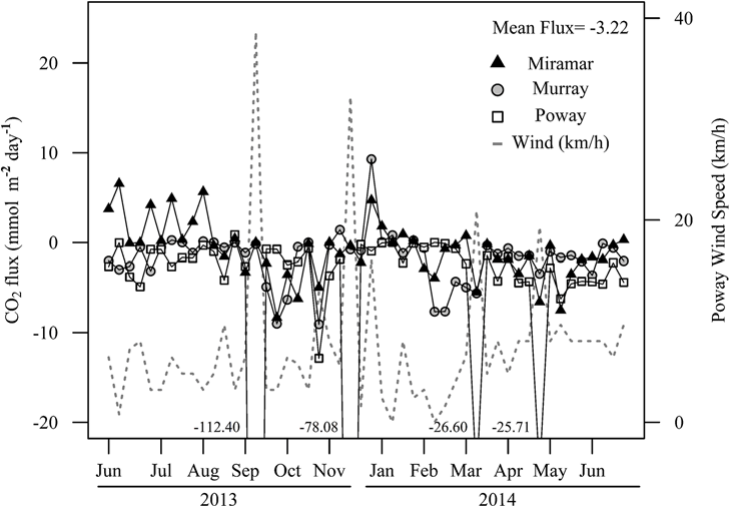
Time series of estimated CO2 flux (mmol m^-2^ day^-1^). The four negative values correspond to CO_2_ flux in Lake Poway that were below the range of the graph during strong wind events.

CO_2_ flux (mmol m^-2^ day^-1^) was estimated to average -3.22 mmol m^-2^ day^-1^ across all three reservoirs (Fig 4). Mean CO_2_ flux for each reservoir are as follows: Murray $\bar{X}$ = -1.755 mmol m^-2^ day^-1^, Miramar $\bar{X}$ = -0.875 mmol m^-2^ day^-1^, Poway $\bar{X}$ = -7.020 mmol m^-2^ day^-1^. Lake Poway had large spikes in CO_2_ uptake on four sampling dates due to high wind speeds (> 5 m s^-1^).

### Phytoplankton Biomass

**Fig 5:**
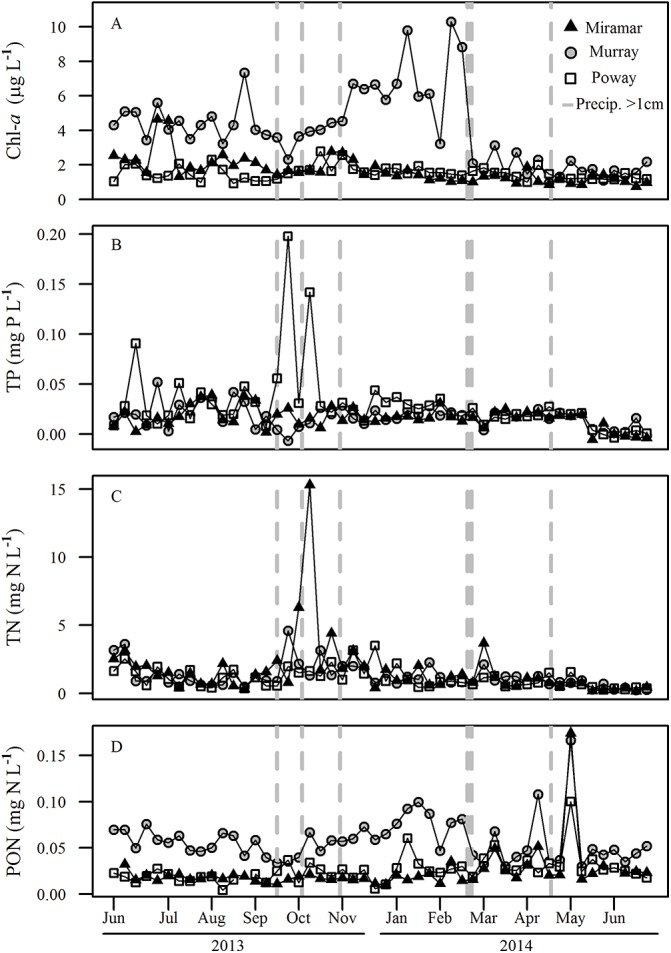
Time series for chlorophyll-*a* (chl-*a*), total phosphorous (TP), total nitrogen (TN), and particulate organic nitrogen (PON) for the three reservoirs. (A) chlorophyll-*a* (μg L^-1^), (B) TP (mg P L^-1^), (C) TN (mg N L^-1^), and (D) PON (mg N L^-1^). The vertical dashed lines represent dates that received >1 cm precipitation. Precipitation during our study period totaled 14.78 cm, and average annual precipitation in San Diego County is 26.26 cm.

Chlorophyll-*a* (μg L^-1^) concentration was consistently highest in Lake Murray ($\bar{X}$ = 4.017 μg L^-1^) and lower in the other two reservoirs (Miramar $\bar{X}$ = 1.684 μg L^-1^; Poway $\bar{X}$ = 1.516 μg L^-1^) and precipitation events did not have an effect on phytoplankton biomass (*p* > 0.05) [Fig 5A]. Chlorophyll-*a* was higher in Lake Murray in the winter than the summer, and higher in summer of 2013 than of 2014. The other two reservoirs showed consistent chlorophyll concentrations between 1–2 μg L^-1^ with no pronounced seasonality. Chlorophyll-*a* was inversely correlated with zooplankton community biomass and mean individual body size in Lake Murray (*r =* -0.323 and *r =* -0.515 respectively) but not in Lake Poway (*r* = 0.001 and *r* = -0.026 respectively) or Lake Miramar (*r* = -0.047 and *r* = -0.072 respectively). The seasonal patterns of pCO_2_ were not mirrored in chlorophyll-*a* within or among reservoirs, and chlorophyll-*a* was not retained as a significant predictor of pCO_2_ in the multiple regression model.

### Total Phosphorous and Nitrogen

Total phosphorous (mg P L^-1^) and total nitrogen (mg N L^-1^) both had low concentrations consistent with oligotrophic conditions, and there were no seasonal trends for either phosphorus or nitrogen in the reservoirs (Fig 5B and 5C). Mean TP concentrations were 0.018 mg P L^-1^ (Murray), 0.016 mg P L^-1^ (Miramar) and 0.030 mg P L^-1^ (Poway). Mean TP across all reservoirs was 0.02 mg P L^-1^. Lake Poway showed two large peaks of total phosphorous (0.14–0.19 mg P L^-1^), which coincided with precipitation events in September–October 2013. Lakes Miramar and Murray both showed peaks in total nitrogen concentration (4.5–15.3 mg N L^-1^) that coincided with precipitation events in September–October 2013 and March 2014. Mean TN concentrations were1.244 mg N L^-1^ (Murray), 1.652 mg N L^-1^ (Miramar) and 1.104 mg N L^-1^ (Poway), with a mean across all reservoirs of 1.33 mg N L^-1^.

### Particulate Organic Nitrogen

Particulate organic nitrogen (PON; mg N L^-1^) concentrations increased during the winter in Lake Murray and showed very high spikes during the spring in all three reservoirs (Fig 5D). Lake Murray consistently had the highest concentrations of PON ($\bar{X}$ = 0.058 mg N L^-1^), while Lakes Poway ($\bar{X}$ = 0.026 mg N L^-1^) and Miramar ($\bar{X}$ = 0.024 mg N L^-1^) had similar, lower concentrations.

### Dissolved and Particulate Organic Carbon

**Fig 6:**
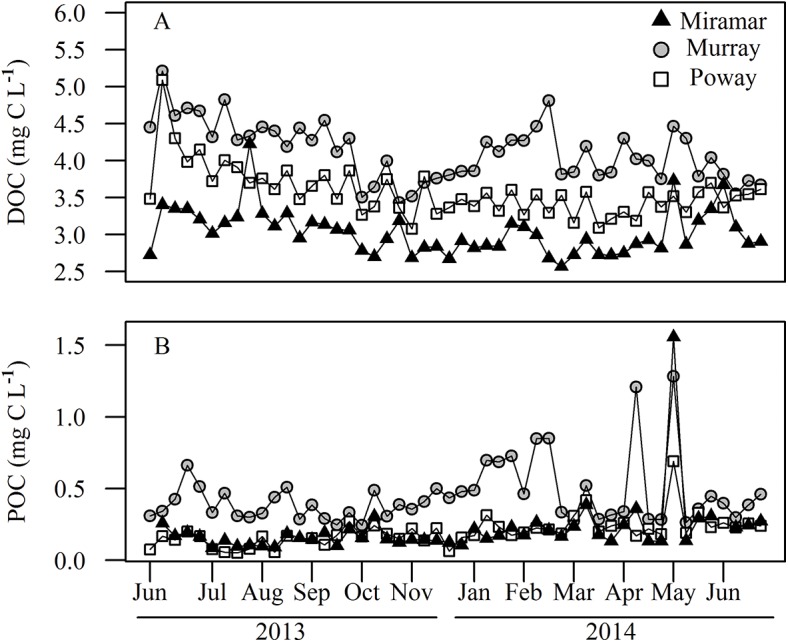
Time series for (A) dissolved organic carbon (DOC; mg C L^-1^), and (B) particulate organic carbon concentrations (POC; mg C L^-1^) for all three reservoirs.

Dissolved organic carbon concentrations (mg C L^-1^) were consistently highest in Lake Murray, the most productive reservoir ($\bar{X}$ = 4.127 mg C L^-1^), lowest in Miramar ($\bar{X}$ = 3.030 mg C L^-1^), and intermediate in Poway ($\bar{X}$ = 3.574 mg C L^-1^) with relatively little seasonal variation in any of the reservoirs. Concentrations declined slightly during the summer and into the winter of 2013, but stayed relatively low during the summer of 2014 (Fig 6A). Particulate organic carbon (POC; mg C L^-1^) concentrations increased during the winter in Lake Murray and showed very high spikes in concentration during the spring in all three reservoirs (Fig 6B). Lake Murray consistently had the highest concentrations of POC ($\bar{X}$ = 0.452 mg C L^-1^) while Lakes Poway ($\bar{X}$ = 0.200 mg C L^-1^) and Miramar ($\bar{X}$ = 0.213 mg C L^-1^) had similar, lower concentrations.

### Bacteria Abundance

**Fig 7:**
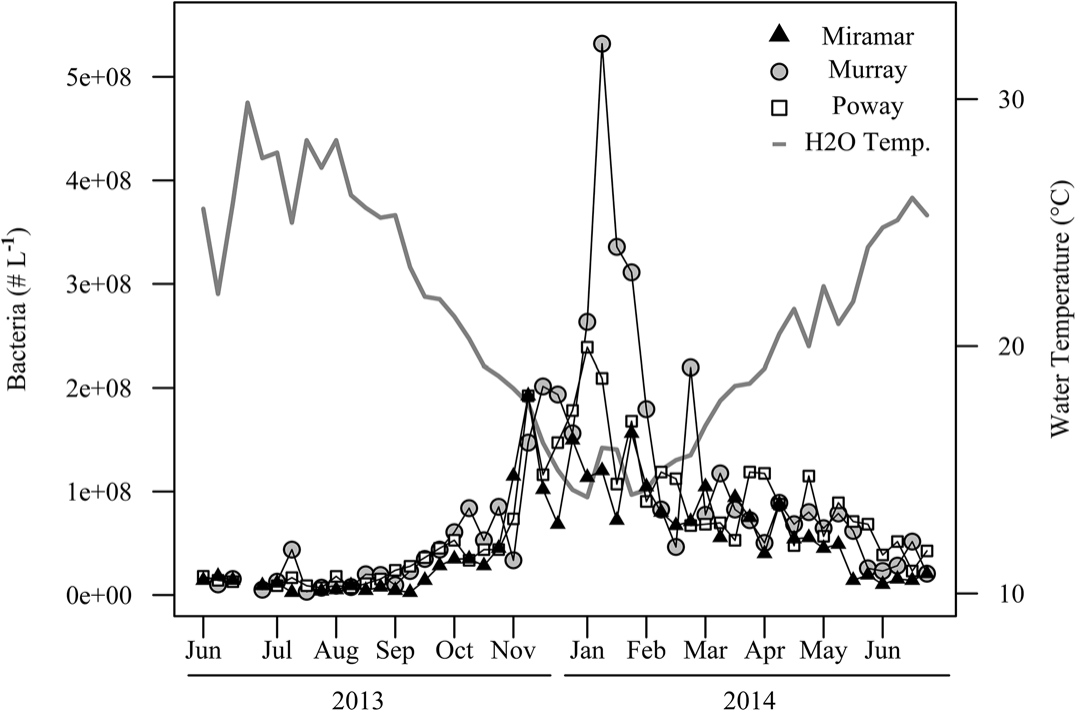
Time series of bacteria abundance (# L^-1^) for all three reservoirs.

Bacteria abundance (# L^-1^) increased during the winter and declined during the summer across all reservoirs (Fig 7). Bacterial abundance was inversely correlated with water temperature (*r* = -0.661).

### Zooplankton Community Biomass

**Fig 8:**
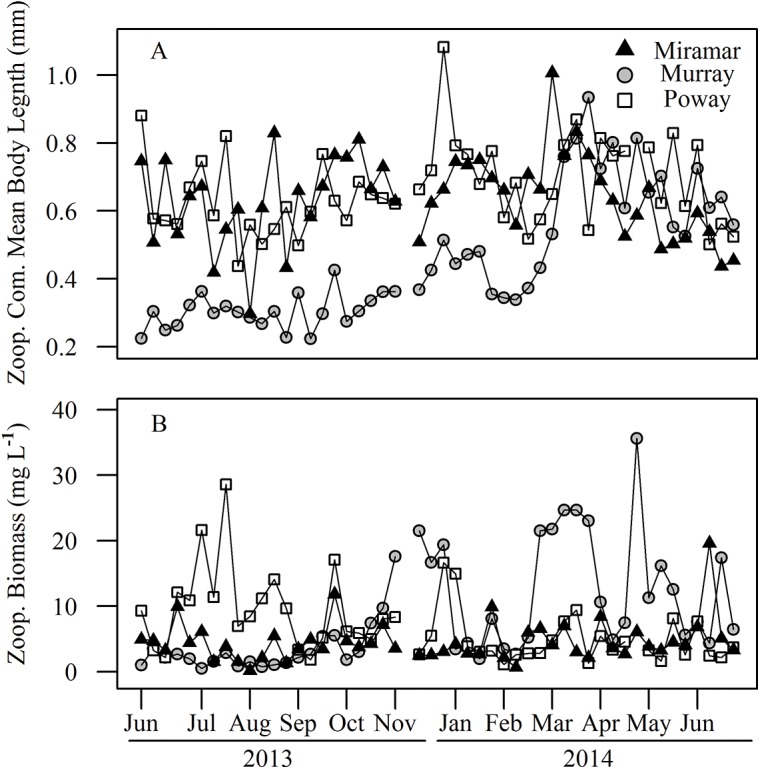
Time series for (A) Zooplankton community mean body length (mm) and (B) community biomass (mg L^-1^) for all three reservoirs.

Lakes Miramar and Poway contained zooplankton communities dominated by larger bodied species than Lake Murray in 2013 (Fig 8A). In the summer of 2014, larger zooplankton increased in abundance in Lake Murray and the mean size of the community reached that of the other two reservoirs during the second year. Chlorophyll concentration declined in Lake Murray at the same time as the increase in abundance of large zooplankton around March of 2014 (Fig 5A).

Zooplankton community biomass (mg L^-1^) was highest in Lake Poway ($\bar{X}$ = 7.028 mg L^-1^) during the summer of 2013 and in Lake Murray ($\bar{X}$ = 8.558 mg L^-1^) in summer of 2014 (Fig 8B). Lake Miramar ($\bar{X}$ = 4.618 mg L^-1^) typically had the lowest zooplankton biomass and the reservoirs differed significantly in zooplankton biomass (*F*_2,140_; p = 0.002). Lake Murray was dominated by small cladocerans and copepods in 2013, while the other two reservoirs had greater numbers of larger *Daphnia* and adult copepods. Large *Daphnia* and copepods increased in density in Lake Murray in spring of 2014.

### Reservoir Stratification

**Fig 9:**
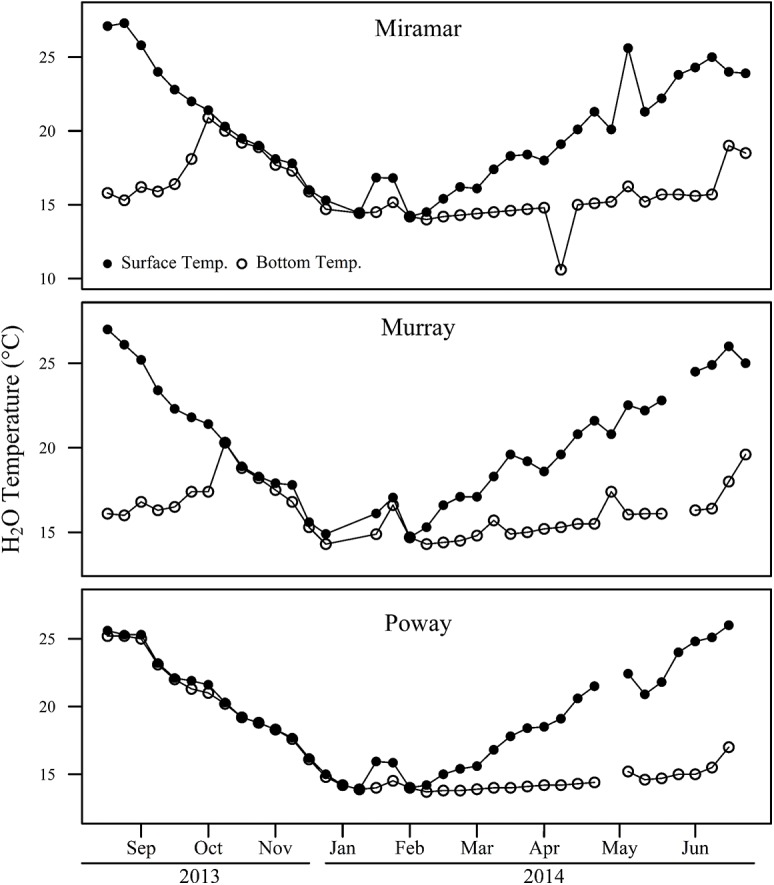
Surface (1 m) and bottom (16–20 m) water temperatures (°C) for each reservoir from Sept 2013-June 2014. The bottom depths for Lakes Poway and Miramar was 20 m and the bottom depth for Lake Murray was 16–17 m. The water columns are stratified when the lines diverge and mixed when the surface and bottom temperatures are the same.

The water columns for Lakes Murray and Miramar were found to be stratified throughout the summer with thermocline depths at 13 m and 19 m respectively, and were not stratified between Oct 2013-Feb 2014 (Fig 9). Lake Poway’s water column was mixed from Sept 2013-Feb 2014 and then remained stratified thereafter with a thermocline depth at 13 m. For all reservoirs, surface water temperatures decreased by about 10°C from summer to winter.

## Discussion

We found that three reservoirs in San Diego, CA were primarily undersaturated with CO_2_ through an entire annual cycle, in contrast with lakes in more heavily vegetated temperate or tropical environments that typically release carbon to the atmosphere [18,43]. This result suggests that biological C uptake through photosynthesis and storage in sediments exceeded microbial respiration at most times of the year. In addition, pCO_2_ increased in the winter and declined in the summer, indicating that seasonal variation is the primary driver of carbon flux. This pattern was similar to that observed by Finlay et al. [44], who showed that alkaline lakes in central North America sequester more CO_2_ in warmer years as a result of reduced CO_2_ accumulation during a shorter ice free season and enhanced chemical conversion of CO_2_ to HCO_3_^-^ and CO_3_^-2^. However, the drinking water reservoirs we study here have pH that is almost always <9, the pH above which chemical uptake becomes significant [44], and are never frozen. Biological uptake is therefore a more likely explanation for the increased CO_2_ uptake that we observed during warmer seasons than chemical conversion. Net primary production is expected to be lowest in the winter when light is limited and day length is short, resulting in higher pCO_2_ concentrations. In addition, surface water inputs are highest in the winter when precipitation occurs, potentially introducing new sources of allochthonous materials. Finally, temporal variation in pCO_2_ was very weakly related to PON and zooplankton biomass, but not chlorophyll-*a* concentration, or the abundance of bacteria. These patterns suggest that bottom-up control of primary production via seasonal variation in light or temperature limitation is the major control on the direction of CO_2_ flux rather than top-down effects of grazing or interactions with microbes.

Studies of pCO_2_ in reservoirs in semi-arid ecosystems or the western U.S. have been carried out on larger spatial scales, but with much less temporal resolution than our study. Soumis et al. [20] found that reservoirs in temperate regions of northern California, Oregon, Washington, and Idaho are primarily sinks for CO_2_, and Therrien et al. [19] concluded that reservoirs in Arizona, New Mexico, and Utah were sources for CO_2_. These studies only sampled lakes and reservoirs on one or two occasions and focused on physico-chemical variables alone as drivers for pCO_2_. They also did not include biological variables, discounting the possible effect of food web dynamics on spatial and temporal variation in pCO_2_. The contrasts between these studies indicate a need for more thorough analysis of the sources of variation in ecosystem metabolism in western reservoirs. Our study demonstrated how seasonal variation in aquatic ecosystem structure is related to spatial and temporal variation in pCO_2_.

Most studies have shown that reservoirs worldwide are primarily sources of CO_2_ to the atmosphere [8,13,23]. These studies have mostly been conducted in highly vegetated systems, such as tropical and temperate forests [14,45] where allochthonous inputs of terrestrial detritus are much higher than in the semi-arid region where our study was conducted [46,47]. Allochthonous organic matter that is respired by heterotrophic microbes increases pCO_2_ and C emissions to the atmosphere [11,22–25]. We observed relatively constant DOC concentrations around 3–5 mg L^-1^, which is low compared to the global mean of around 7 mg L^-1^ [43]. Barros et al. [14] concluded from a global survey that C-flux in hydroelectric reservoirs is related to age and latitude; however, their data are highly clustered in the tropics (<22°) and temperate zone (>45°), with almost no samples in between (see their Fig 1). Our results indicate that without high levels of decomposition associated with large inputs of organic matter, primary production may exceed heterotrophic respiration and lead to sequestration of atmospheric C in reservoirs in the sparsely vegetated and semi-arid sub-tropics. Thus, organic matter may be deposited in the sediments of reservoirs for long periods of time until either water from the bottom of the reservoir is released or upwelling occurs.

We measured in situ chlorophyll-*a* concentration as an indicator of the standing biomass of phytoplankton. However, chlorophyll-*a* is not a good indicator of whole ecosystem primary production as benthic autotrophs (macrophytes and periphyton) play an important role, and because changes in growth may not be reflected by changes in biomass. We did not measure net production directly, however it is likely to be higher in the summer when temperatures are warmer and days are longer, although we saw no increase in chlorophyll-*a* concentrations. Highly eutrophic lakes tend to function as carbon sinks due to high biological C uptake [6], while dystrophic lakes emit CO_2_ due to respiration of allochthonous organic matter [2]. However, seasonal and spatial variability in chlorophyll-*a* and DOC concentrations were poor predictors of pCO_2_ in our study, likely because variation in production within or among our reservoirs was small compared to the global range where chlorophyll-*a* concentrations can exceed 1000 *μ*g L^-1^. We saw maximum chlorophyll-*a* concentrations around 10 *μ*g L^-1^, indicating that the reservoirs were always within the oligotrophic-mesotrophic range. The lack of association between chlorophyll-*a* and pCO_2_ indicates either that other chemical or biological processes regulate carbon dynamics in these reservoirs, or that annual variation in ecosystem respiration is not reflected in phytoplankton standing stock. However, our results do show that CO_2_ is most depleted in the water column in the summer when ecosystem production is expected to be greatest [44]. Biological uptake may therefore play a role in the undersaturation of CO_2_ in these reservoirs.

Although variation in chlorophyll-*a* was not associated with pCO_2_, we observed seasonal changes in chlorophyll-*a* concentrations across the reservoirs. In Lake Murray, concentrations were consistent until zooplankton community biomass and mean body length size for zooplankton increased in 2014, accompanied by a rapid decline in chlorophyll-*a* concentrations. This pattern suggests that an increase in abundance of large bodied zooplankton and elevated grazing pressure resulted in lower biomass of phytoplankton. In contrast, Lakes Miramar and Poway did not exhibit any seasonal or interannual changes in chlorophyll-*a* concentrations or zooplankton abundance or size structure. Although Lake Murray was more eutrophic than the other two for most of the study, it did not show consistently lower pCO_2_ as we would expect if variation in pelagic primary production was the major factor controlling pCO_2._ Benthic production and decomposition, which contribute greatly to whole system metabolic balance but were not measured in our study, may explain this disparity

Increases in pCO_2_ during the winter may have resulted from heterotrophic respiration exceeding photosynthesis by phytoplankton during periods of greater light limitation and lower temperatures. Microbes that degrade organic matter, including bacteria, also increased during colder months and may have contributed to higher respiration and greater pCO_2_ [15]. During the winter, the water column became less stratified, both in temperature and dissolved oxygen concentrations. With a more homogenous water column, sediment and buried organic matter and nutrients from the bottom of the reservoirs could be resuspended to the surface via mixing and potentially decomposed by pelagic bacteria, contributing to higher pCO_2_.

Zooplankton community biomass did not show a consistent seasonal trend across all reservoirs, but there were large changes in community structure within each lake. These changes over time were not strongly correlated with chlorophyll-*a*, except in Lake Murray where increases in zooplankton biomass and mean size in the summer of 2014 were associated with declining chlorophyll-*a* concentrations. This suggests that changes in predation or other factors caused shifts in the zooplankton community of Lake Murray with cascading effects on phytoplankton biomass. The mean lengths for zooplankton were consistent over time in Lakes Miramar and Poway, but increased in Lake Murray during the spring. The cause of the increase in zooplankton size in Lake Murray is unclear, but its association with a sharp decline in chlorophyll-*a* indicates a strong top-down effect of zooplankton on primary producers, even though this shift had no apparent effect on estimated CO_2_ flux.

The concentrations of DOC in the reservoirs we studied were uncorrelated with pCO_2_, in contrast with most studies estimating CO_2_ flux in lakes and reservoirs [3,43,48]. We may have observed no association between DOC and pCO_2_ because the concentrations were low compared to the global range [43]. In addition, we saw little seasonality in DOC in contrast with temperate regions with deciduous vegetation that produces an annual leaf drop in the fall [49]. Our bulk measure does not distinguish between DOC of autochthonous vs. allochthonous origin. The quality of terrestrial DOC as substrate for microbes is much lower than that produced by aquatic producers, and this difference may be important to determining pCO_2_ [50]. However, our results show no evidence that spatial or seasonal variations in DOC concentration are a significant for determining pCO_2_ in the studied reservoirs.

Ongoing changes in climate are likely to produce declines in the availability and quality of freshwater resources worldwide. This trend is already increasing pressure to construct impoundments to capture more surface water for human uses in semi-arid regions such as Southern California. Thus, it is essential to understand how water storage contributes to the global carbon cycle and whether feedbacks are likely to accelerate or dampen ongoing emissions of greenhouse gases to the atmosphere. We found that three of the 24 major reservoirs in San Diego County are undersaturated in CO_2_ throughout the year. Increased construction of reservoirs to store water is therefore unlikely to provide a significant new source of CO_2_ to the atmosphere. Our data also indicate that seasonal changes in photosynthesis, likely driven by light availability and temperature, drive pCO_2_ dynamics over time more than the abundance of pelagic primary producers, their zooplankton consumers, or the concentration of DOC. Our results provide a framework for forecasting the impacts of reservoir construction in the arid southwest of North America on the regional carbon budget, and for understanding the ecological and environmental constraints on CO_2_ uptake in these anthropogenic ecosystems.

## References

[1] Schindler DE, Carpenter SR, Cole JJ, Kitchell JF, Pace ML. Influence of Food Web Structure on Carbon Exchange Between Lakes and the Atmosphere. Science. 1997;277: 248–251. 10.1126/science.277.5323.248

[2] Prairie YT, Bird DF, Cole JJ. The summer metabolic balance in the epilimnion of southeastern Quebec lakes. Limnol Oceanogr. 2002;47: 316–321.

[3] Sobek S, Algesten G, Bergström A-K, Jansson M, Tranvik LJ. The catchment and climate regulation of pCO2 in boreal lakes. Glob Change Biol. 2003;9: 630–641. 10.1046/j.1365-2486.2003.00619.x

[4] Lapierre J-F, Guillemette F, Berggren M, del Giorgio PA. Increases in terrestrially derived carbon stimulate organic carbon processing and CO2 emissions in boreal aquatic ecosystems. Nat Commun. 2013;4 10.1038/ncomms3972 24336188

[5] Larmola T, Alm J, Juutinen S, Martikainen PJ, Silvola J. Ecosystem CO2 exchange and plant biomass in the littoral zone of a boreal eutrophic lake. Freshw Biol. 2003;48: 1295–1310. 10.1046/j.1365-2427.2003.01079.x

[6] Pacheco FS, Roland F, Downing JA. Eutrophication reverses whole-lake carbon budgets. Inland Waters. 2013;4: 41–48.

[7] Atwood TB, Hammill E, Greig HS, Kratina P, Shurin JB, Srivastava DS, et al Predator-induced reduction of freshwater carbon dioxide emissions. Nat Geosci. 2013;6: 191–194. 10.1038/ngeo1734

[8] Cole JJ, Prairie YT, Caraco NF, McDowell WH, Tranvik LJ, Striegl RG, et al Plumbing the Global Carbon Cycle: Integrating Inland Waters into the Terrestrial Carbon Budget. Ecosystems. 2007;10: 172–185. 10.1007/s10021-006-9013-8

[9] Molot LA, Dillon PJ. Storage of terrestrial carbon in boreal lake sediments and evasion to the atmosphere. Glob Biogeochem Cycles. 1996;10: 483–492. 10.1029/96GB01666

[10] Kelly CA, Rudd JWM, Bodaly RA, Roulet NP, St.Louis VL, Heyes A, et al Increases in Fluxes of Greenhouse Gases and Methyl Mercury following Flooding of an Experimental Reservoir. Environ Sci Technol. 1997;31: 1334–1344. 10.1021/es9604931

[11] Tranvik LJ. Availability of dissolved organic carbon for planktonic bacteria in oligotrophic lakes of differing humic content. Microb Ecol. 1988;16: 311–322. 10.1007/BF02011702 24201716

[12] Dean WE, Gorham E. Magnitude and significance of carbon burial in lakes, reservoirs, and peatlands. Geology. 1998;26: 535–538. 10.1130/0091-7613(1998)026<0535:MASOCB>2.3.CO;2

[13] St. Louis VL, Kelly CA, Duchemin É, Rudd JWM, Rosenberg DM. Reservoir Surfaces as Sources of Greenhouse Gases to the Atmosphere: A Global Estimate Reservoirs are sources of greenhouse gases to the atmosphere, and their surface areas have increased to the point where they should be included in global inventories of anthropogenic emissions of greenhouse gases. BioScience. 2000;50: 766–775. 10.1641/0006-3568(2000)050[0766:RSASOG]2.0.CO;2

[14] Barros N, Cole JJ, Tranvik LJ, Prairie YT, Bastviken D, Huszar VLM, et al Carbon emission from hydroelectric reservoirs linked to reservoir age and latitude. Nat Geosci. 2011;4: 593–596. 10.1038/ngeo1211

[15] del Giorgio PA, Cole JJ, Cimbleris A. Respiration rates in bacteria exceed phytoplankton production in unproductive aquatic systems. Nature. 1997;385: 148–151. 10.1038/385148a0

[16] Andersen M. An Overview of U.S. Reservoir GHG Emissions Studies and Preliminary Automated Sampling Results for Lake Oroville [Internet]. The Climate Registry: State of the Science: GHG Emissions from Hydro Reservoirs; A Dialogue with Experts; 2009 Sep 17; Montreal, QC. Available: http://www.theclimateregistry.org/downloads/2009/05/GHG-emissions-from-reservoirs-in-the-USA-Mark-Andersen-DWR-California.pdf

[17] Lazzarino JK, Bachmann RW, Hoyer MV, Jr DEC. Carbon dioxide supersaturation in Florida lakes. Hydrobiologia. 2009;627: 169–180. 10.1007/s10750-009-9723-y

[18] Roehm CL, Prairie YT, del Giorgio PA. The pCO2 dynamics in lakes in the boreal region of northern Québec, Canada. Glob Biogeochem Cycles. 2009;23: GB3013 10.1029/2008GB003297

[19] Therrien J, Tremblay A, Jacques RB. CO2 Emissions from Semi-Arid Reservoirs and Natural Aquatic Ecosystems In: Tremblay DA, Varfalvy DL, Roehm DC, Garneau DM, editors. Greenhouse Gas Emissions—Fluxes and Processes. Springer Berlin Heidelberg; 2005 pp. 233–250. Available: http://link.springer.com/chapter/10.1007/978-3-540-26643-3_10

[20] Soumis N, Duchemin É, Canuel R, Lucotte M. Greenhouse gas emissions from reservoirs of the western United States. Glob Biogeochem Cycles. 2004;18: GB3022 10.1029/2003GB002197

[21] Morales-Pineda M, Cózar A, Laiz I, Úbeda B, Gálvez JÁ. Daily, biweekly, and seasonal temporal scales of pCO2 variability in two stratified Mediterranean reservoirs. J Geophys Res Biogeosciences. 2014;119: 2013JG002317 10.1002/2013JG002317

[22] Jansson M, Bergström A-K, Blomqvist P, Drakare S. Allochthonous organic carbon and phytoplankton/bacterioplankton production relationships in lakes. Ecology. 2000;81: 3250–3255. 10.1890/0012-9658(2000)081[3250:AOCAPB]2.0.CO;2

[23] Sobek S, Tranvik LJ, Cole JJ. Temperature independence of carbon dioxide supersaturation in global lakes. Glob Biogeochem Cycles. 2005;19: GB2003 10.1029/2004GB002264

[24] del Giorgio PA, Peters RH. Patterns in Planktonic P:R Ratios in Lakes: Influence of Lake Trophy and Dissolved Organic Carbon. Limnol Oceanogr. 1994;39: 772–787.

[25] Cole JJ, Pace ML, Carpenter SR, Kitchell JF. Persistence of Net Heterotrophy in Lakes during Nutrient Addition and Food Web Manipulations. Limnol Oceanogr. 2000;45: 1718–1730. 10.4319/lo.2000.45.8.1718

[26] del Giorgio PA, Cole JJ, Caraco NF, Peters RH. Linking planktonic biomass and metabolism to net gas fluxes in northern temperate lakes. Ecology. 1999;80: 1422–1431. 10.1890/0012-9658(1999)080[1422:LPBAMT]2.0.CO;2

[27] Robbins LL, Hansen ME, Kleypas JA, Meylan SC. CO2calc—A user-friendly seawater carbon calculator for Windows, Max OS X, and iOS (iPhone): U.S. Geological Survey Open-File Report 2010–1280, 17 p. [Internet]. 2010 [cited 19 Sep 2014]. Available: http://pubs.usgs.gov/of/2010/1280/

[28] Millero FJ. The thermodynamics of the carbonate system in seawater. Geochim Cosmochim Acta. 1979;43: 1651–1661. 10.1016/0016-7037(79)90184-4

[29] Dickson AG. Thermodynamics of the dissociation of boric acid in synthetic seawater from 273.15 to 318.15 K. Deep Sea Res Part Oceanogr Res Pap. 1990;37: 755–766. 10.1016/0198-0149(90)90004-F

[30] Vincent WF. Mechanisms of Rapid Photosynthetic Adaptation in Natural Phytoplankton Communities. I. Redistribution of Excitation Energy Between Photosystems I and Ii1. J Phycol. 1979;15: 429–434. 10.1111/j.1529-8817.1979.tb00715.x

[31] Vincent WF. Mechanisms of Rapid Photosynthetic Adaptation in Natural Phytoplankton Communities. Ii. Changes in Photochemical Capacity as Measured by Dcmu-Induced Chlorophyll Fluorescence1. J Phycol. 1980;16: 568–577. 10.1111/j.1529-8817.1980.tb03075.x

[32] Vyhnalek ZF V. In vivo fluorescence of chlorophyll A: Estimation of phytoplankton biomass and activity in Rimov Reservoir (Czech Republic). Water Sci Technol. 1993;28: 29–33.

[33] Porter KG, Feig YS. The use of DAPI for identifying and counting aquatic microflora1. Limnol Oceanogr. 1980;25: 943–948. 10.4319/lo.1980.25.5.0943

[34] Rasband WS. ImageJ. In: ImageJ, U.S. National Institutes of Health, Bethesda, Maryland, USA [Internet]. 2014 1997 [cited 19 Sep 2014]. Available: http://imagej.nih.gov/ij/

[35] Dumont HJ, Velde IV de, Dumont S. The dry weight estimate of biomass in a selection of Cladocera, Copepoda and Rotifera from the plankton, periphyton and benthos of continental waters. Oecologia. 1975;19: 75–97. 10.1007/BF00377592 28308833

[36] Bottrell H, Duncan A, Gliwicz ZM, Grygierek E, Herzig A, Hillbricht-Ilkowska A, et al A review of some problems in zooplankton production studies. Norw J Zool. 1976;24: 419–456.

[37] Rosen RA. Length-Dry Weight Relationships of Some Freshwater Zooplanktona. J Freshw Ecol. 1981;1: 225–229. 10.1080/02705060.1981.9664034

[38] McCauley E. The estimation of the abundance and biomass of zooplankton samples. A manual on the methods for the assessment of secondary productivity in fresh waters. 1984 pp. 228–265.

[39] Malley DF, Lawrence SG, MacIver MA, Findlay WJ. Range of Variation in Estimates of Dry Weight for Planktonic Crustacea and Rotifera from Temperate North American Lakes. Fisheries and Oceans, Canada; 1989.

[40] Watkins J, Rudstam L, Holeck K. Length-weight regressions for zooplankton biomass calculations–A review and a suggestion for standard equations. 2011; Available: http://ecommons.library.cornell.edu/handle/1813/24566

[41] Bartoń K. MuMIn: Multi-model inference. R package version 1.10.5. http://CRAN.R-project.org/package=MuMIn [Internet]. 2014. Available: http://CRAN.R-project.org/package=MuMIn

[42] R Core Team. R: A language and environment for statistical computing [Internet]. Vienna, Austria: R Foundation for Statistical Computing; 2015 Available: http://www.R-project.org/.

[43] Sobek, Tranvik LJ, Prairie YT, Kortelainen P, Cole JJ. Patterns and regulation of dissolved organic carbon: An analysis of 7,500 widely distributed lakes. Limnol Oceanogr. 2007;52: 1208–1219.

[44] Finlay K, Vogt RJ, Bogard MJ, Wissel B, Tutolo BM, Simpson GL, et al Decrease in CO2 efflux from northern hardwater lakes with increasing atmospheric warming. Nature. 2015;519: 215–218. 10.1038/nature14172 25731167

[45] Marotta H, Duarte CM, Sobek S, Enrich-Prast A. Large CO2 disequilibria in tropical lakes. Glob Biogeochem Cycles. 2009;23: GB4022 10.1029/2008GB003434

[46] Howarth RW, Schneider R, Swaney D. Metabolism and organic carbon fluxes in the tidal freshwater Hudson River. Estuaries. 1996;19: 848–865. 10.2307/1352302

[47] Peixoto RB, Marotta H, Enrich-Prast A. Experimental evidence of nitrogen control on pCO2 in phosphorus-enriched humic and clear coastal lagoon waters. Front Microbiol. 2013;4 10.3389/fmicb.2013.00011 PMC3565232 23390422

[48] Lennon JT. Experimental evidence that terrestrial carbon subsidies increase CO2 flux from lake ecosystems. Oecologia. 2004;138: 584–591. 10.1007/s00442-003-1459-1 14689297

[49] Cottingham KL, Narayan L. Subsidy quantity and recipient community structure mediate plankton responses to autumn leaf drop. Ecosphere. 2013;4: art89. 10.1890/ES13-00128.1

[50] McKnight DM, Boyer EW, Westerhoff PK, Doran PT, Kulbe T, Andersen DT. Spectrofluorometric characterization of dissolved organic matter for indication of precursor organic material and aromaticity. Limnol Oceanogr. 2001;46: 38–48. 10.4319/lo.2001.46.1.0038

